# Investigating the Diversity of Marine Bacteriophage in Contrasting Water Masses Associated with the East Australian Current (EAC) System

**DOI:** 10.3390/v12030317

**Published:** 2020-03-16

**Authors:** Amaranta Focardi, Martin Ostrowski, Kirianne Goossen, Mark V. Brown, Ian Paulsen

**Affiliations:** 1 Department of Molecular Sciences, Macquarie University, 4 Wally’s Walk, Sydney, NSW 2109, Australia; amaranta.focardi@mq.edu.au; 2 Climate Change Cluster, University of Technology Sydney, 123 Broadway, Sydney, NSW 2007, Australia; martin.ostrowski@uts.edu.au; 3 CSIRO Oceans and Atmosphere, Castray Esplanade, Hobart, TAS 7001, Australiamark.v.brown@newcastle.edu.au (M.V.B.); 4 School of Environmental and Life Sciences, University of Newcastle, University Dr, Callaghan, NSW 2308, Australia

**Keywords:** bacteriophage, metagenome, metaviromes, east Australian current, Tasman Sea

## Abstract

Virus- and bacteriophage-induced mortality can have a significant impact on marine productivity and alter the flux of nutrients in marine microbial food-webs. Viral mediated horizontal gene transfer can also influence host fitness and community composition. However, there are very few studies of marine viral diversity in the Southern Hemisphere, which hampers our ability to fully understand the complex interplay of biotic and abiotic factors that shape microbial communities. We carried out the first genetic study of bacteriophage communities within a dynamic western boundary current (WBC) system, the east Australian current (EAC). Virus DNA sequences were extracted from 63 assembled metagenomes and six metaviromes obtained from various depths at 24 different locations. More than 1700 bacteriophage genomic fragments (>9 kbps) were recovered from the assembled sequences. Bacteriophage diversity displayed distinct depth and regional patterns. There were clear differences in the bacteriophage populations associated with the EAC and Tasman Sea euphotic zones, at both the taxonomic and functional level. In contrast, bathypelagic phages were similar across the two oceanic regions. These data provide the first characterisation of viral diversity across a dynamic western boundary current, which is an emerging model for studying the response of microbial communities to climate change.

## 1. Introduction

Viruses are the most abundant entity in the marine environment. Their concentration can reach as high as 10^7^ mL^−1^ and they may outnumber bacteria by more two orders of magnitude [1]. The majority of oceanic viruses infect bacteria and are termed bacteriophages [2]. Bacteriophages (and viruses in general) have a considerable influence on the ecology and the biogeochemical cycles of the ocean [1,3]. Viral-induced mortality can influence the flux of nutrients in microbial food-webs through the release of “new” dissolved organic matter (DOM) [4]. It is estimated that marine viruses promote the release of up to 145 gigatons of carbon per year [5] and therefore represent a major route of C-recycling. The DOM released via viral-induced mortality can either fuel microbial growth through the recycling of nutrients that are limited in the ocean [6,7], or enhance the efficiency of the carbon pump [8] by enhancing the formation of fast sinking aggregates and colloidal particles [8,9]. Moreover, viruses can promote genetic variation of their host species via horizontal gene transfer [10] and alter ecosystem productivity through the expression of specific viral-encoded auxiliary metabolic genes (AMGs) [11]. AMGs are widespread in bacteriophage and are believed to encode functions that sustain the host metabolism during infection. In cyanophages for example, the core photosystem II genes *psbA* and *psbD* [12,13] play a role in maintaining active photosynthetic electron transport, in order to accumulate energy for phage production. This is particularly important under high light conditions, where the surplus of energy from the upregulation of bacteriophage *psbA* can support an increased translation rate and is responsible for a shortening of the lytic cycle [14]. Other examples of AMGs are those involved in carbon metabolism, like *cp12* and *talC* [13,15,16] that inhibit the Calvin cycle and redirect carbon flux to the pentose phosphate pathway [17] to promote deoxynucleotide biosynthesis.

Despite the importance of bacteriophage in the marine environment there is still a paucity of information on the diversity of viruses in the southern hemisphere. Here, we carried out the first study of the bacteriophage diversity in the oceanic region associated with the Pacific Ocean western boundary current (WBC), the east Australian current (EAC). The EAC is an important model system to investigate the biological impacts of climate change [18,19,20]. It supports the poleward transport of heat and nutrient poor waters into temperate latitudes [21,22] and has played a significant role in shifting the biogeographic boundaries of regional ecological provinces in the Tasman Sea [23]. Previous studies have underlined how the EAC and Tasman Sea maintain distinct microbial assemblages associated with the different oceanographic characteristic of each system [24,25]. While the balance between microbial dispersal and selection in this dynamic region is complex, we hypothesized that viral-mediated mortality is playing a pivotal role in community turnover, and hence may be a driver of the emergent patterns of microbial diversity across this region. However, there is no information about the identity and diversity of the main viral groups, and especially those infecting primary producers. This gap in knowledge limits our ability to fully understand the factors that govern microbial community structure, which influences present and future productivity in a region undergoing significant climate-related change [18,19,20,21].

Here, we present the first study of the genetic diversity of viral communities in both the euphotic and aphotic zone of the EAC and the Tasman Sea, from samples that cover nine degrees of latitude (28°–37° S). Viral genome fragments were assembled from metagenomic contigs, derived from both the microbial (>0.2 µm dia.) and the viral size fractions (<0.2 µm dia.) These assemblies represent the novel genetic diversity of the marine virus present in Southern Hemisphere waters.

## 2. Materials and Methods

### 2.1. Sample Collection

**Figure 1:**
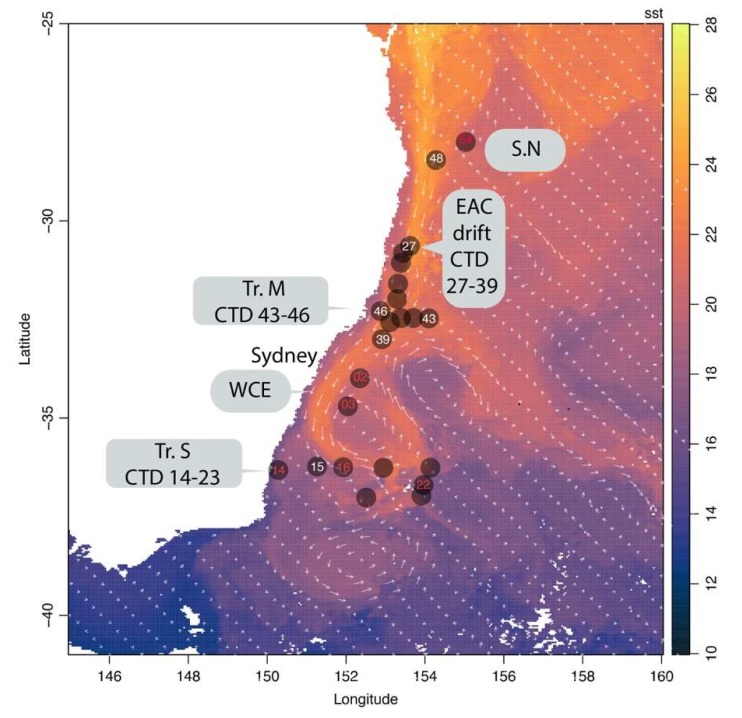
Location of the sampling sites along the east coast of Australia: Transect South (Tr.S), Transect middle (Tr.M), the Lagrangian drift along the EAC (EAC drift), the northern samples (SN) and the samples collected inside a warm core eddy (WCE). Data for the 8-day composite average surface seawater temperature on a 4 km resolution were obtained from the Australian Ocean Data Network portal (https://portal.aodn.org.au/) for the period September 2016. Sampling sites and transects are highlighted. Colours of the CTD deployments are related to the availability of metagenomes for either the microbial fraction (white) (>0.2 µm) or the microbial (>0.2 µm) and viral fraction (<0.2 µm) (red).

Samples were collected from 26 stations during an oceanographic expedition on board the R/V Investigator (IN2016_v04) in September 2016. Sample sites were located on two shore-normal longitudinal transects at the southern (Tr_S: 36.9° S, 150.9°−154.25° E) and middle (Tr_M: 32.4° S, 152.87°–154.10° E) section of the voyage, as well as a Lagrangian drift along the east Australian current (30.65°–32.99° S), and two sites in the northern sector of the voyage (Tr_N: 28° S–155° E) (Figure 1) (Supplementary Table S1). For each site, water from specific depths up to 4000 m was sampled, using a rosette equipped with 24 × 12 L Niskin bottles and a CTD fitted with additional optical sensors (Fluorometry, Oxygen). Sixty samples were collected for the microbial fraction (>0.2 µm) and six samples for the viral fraction (<0.2 µm) (Supplementary Table S1).

For the larger fraction, 2 L of seawater were filtered through a 0.2 µm dia. pore size Polyethersulfone (PES) filter (Sterivex, Millipore, Burlington, MA, USA) and stored at −80 °C [24]. Usually 3–4 depths were sampled at each station (2–3 in the euphotic zone and the others in the aphotic zone). For the viral fraction, 20 L of 0.2 µm filtered seawater was treated with 1 mL of iron chloride (10 g/L) for 1 h [26,27], to flocculate the viral particles at room temperature. The flocculate was collected by filtration on a 0.8 µm dia. pore size polycarbonate filter (147 mm dia., ATTP14250, Millipore), and stored at 4 °C until subsequent processing (within two months of sampling). A total of 5 viral samples were collected from the surface (~2 m depth), and one sample was collected at 3000 m. Two of the surface samples were collected in a warm core eddy (CTD02 and CTD03).

### 2.2. DNA Extraction

For the viral fraction, DNA was extracted using a modification of the iron chloride flocculation protocol [26]. Briefly, the viral particle concentrate was resuspended in ascorbate buffer (5.0 mL 0.1 M EDTA-0.2 M MgCl_2_-0.2 M) [26] on a rotary shaker overnight at 4 °C and, then concentrated using an Amicon column (30 kDa) at 4 °C and 1000× *g*, until the desired volume (1 mL) was reached. After resuspension, samples were treated with DNAase 100 U mL^−1^ (Roche, Basel, Switzerland) in a reaction buffer at 37 °C for 1 h [27]. The DNAase was inactivated with an equimolar concentration of EDTA/EGTA (final concentration 0.1 M), used to chelate the metal ions. DNA was subsequently extracted using the AMPvir kit (Qiagen, Hilden, Germany), following the protocol recommendation. Library preparation for NGS was carried out using the Nextera XT kit for low DNA yield samples and sequenced on an Illumina Miseq platform at the Ramaciotti Centre for Genomics, UNSW, Australia. DNA for sequencing the microbial fraction were extracted and sequenced, as part of the Australian Microbiome Initiative (formerly the Australian Marine Microbes Project), following their standard protocol [24].

### 2.3. Metagenome Analysis

The quality of both forward and reverse sequence reads were assessed with FastQC [28]. Adaptors and low quality reads were removed with Trimmomatic-0-3.36 [29], using default parameters, and trimmed reads were assembled with Spades (v 3.10.1) using the careful option [30], in line with the recommendations of Nishimura et al., 2017 [31]. The average length of assembled reads spanned from a minimum length of 300 to a max of 198,000 nt. DNA from each sample was assembled separately and then contigs from all assemblies were pooled together and dereplicated with CD-HIT [32], using a 98% similarity threshold; a total of 22,701,531 contigs were identified. Putative bacteriophage contigs were then identified using Virsorter v.1.05 [33]. A total of 26,560 contigs, ranging between 300 and 197,000 nt, that were classified into either of the two highest confidence prediction categories in Virsorter (categories 1 and 2), were retained for the further analysis of bacteriophage assemblage diversity. Virsorter was optimized for the detection of bacterial viruses and therefore it does not perform well in detecting eukaryotic viruses. MetaGeneMark2 [34] was then used to identify open reading frames.

The raw data used in this work have been deposited in the Sequence Read Archive (Bioproject number: microbial PRJNA385736, virus PRJNA516152); the accession number for each sample is available in the Supplementary Table S1.

### 2.4. Taxonomy of Viral and Prokaryotics Contigs

Taxonomy was assigned using Kaiju (greedy mode) [35] against the NCBI virus database (December 2019), with an e-value cut-off set to 0.05. Similarities to previously sequenced viral DNA fragments from metagenomic studies were assessed with a BLASTn search against the IMG/VR database (2018). Viral fragments were considered similar if they shared >50% of the genes with ≥ 70% of nt similarity. This relaxed cut-off was chosen in order to identify sequences distantly related to previously sequenced and assembled bacteriophage contigs in the IMG/VR database [36]. Viral contigs were grouped into four categories based on the number of genes shared at >70% nucleotide identity with their closest related sequence: novel bacteriophage (N) (<20% shared genes), low similarity (LS) (20–60% shared genes), similar (S) (60–80% shared genes), known phage (K) (>80% shared genes).

Community profiles for archaea and bacteria were estimated from SSU rRNA genes reconstructed from shotgun metagenomes, using phyloFlash (v3.0) [37] and blast against the GTDB database for taxonomic annotation [38]. The taxonomic composition of all 63 (>0.22 µm) metagenomes was converted into an abundance matrix using phyloFlash_compare.pl., following a normalisation step based on read counts for each sample. To calculate the relative abundance of SSU rRNA per sample, the number of mapped reads to each SSU rRNA were divided by the total reads number (per sample) (Supplementary Table S2).

### 2.5. Identification of Bacteriophage Contigs

In order to better understand bacteriophage distribution in Australian waters, we selected contigs >9 kbps from the total dataset. The 9 kbps cut-off was chosen based on the genome size (10 kb genome) of a recent discovered and, highly widespread, family of viruses (*Autolykiviridae*) [39,40], and considered to be within the smallest bacteriophage in the oceanic environment. These contigs underwent a second quality control step, and only those with at least 6 hits to a viral protein family (VPFs, HMM modules from IMG/VR) were retained. This approach selected a final subset of 1770 contigs. Filtered reads from each metagenome or metavirome were mapped against the final viral contigs database using bowtie2 [41]. The relative coverage of each contig from each of the different sites was calculated by dividing the number of mapped reads per contig by the total number of filtered reads in each sample.

### 2.6. Analysis of Functional Gene Profiles for Each Site and Bacteriophage Lifestyle

To characterise the functional gene diversity, the genome-centric approach was followed by a gene-centric approach. Genes from all the 26,560 contigs were annotated with Interproscan and eggNOG5 with an evalue cut-off of <10^−5^. The function was assigned to a total of almost 37,000 genes (Supplementary Table S3). Similar to the approach used by Coutinho et al., 2017 [42], the functional profile for each sample was characterised by summing the relative abundance of each pfam proportionally to the relative abundance (normalized) of the contigs in which it was found. For example, if contig A contained gene 1 and gene 2 and its abundance was 10 in sample A and 2 in sample B, then the functional profile for sample A was calculated as gene 1 multiplied by ten plus gene 2 multiplied by 10, while for sample B, genes were multiplied by 2.

To better characterize the predominance of either temperate or lytic phage in the different environments, we looked for the distribution of specific lysogenic marker genes, in particular the distribution of bacteriophage integrases. We used a curated set of 210 refined integrase genes that specifically belong to bacteriophage, annotated from NCBI, to generate a marker gene database. We used this database to identify integrase genes via a BLASTn search (evalue <10^−5^) in the gene catalogue from the complete contigs set, including both microbial and viral contigs.

### 2.7. Statistical Analysis

A statistical analysis was performed in R v3.4.1 [43]. To better understand bacteriophage regional distribution, the environmental abundance of each contig was inferred from the number of metagenomic reads mapped from each sample (Supplementary Table S2). These abundances were rarefied and square root transformed and the patterns of viral diversity in each sample, and were clustered based on similarity (Bray–Curtis), with clustering analysis using SIMPROF (clustsig) [44]. The significance of the cluster was then tested with ADONIS (Vegan) [45]. Contigs that significantly contributed to the dissimilarity between cluster and the a-priori designated “Oceanic provinces” were identified using SIMPER (similarity percentage). Principal component analysis (Vegan) identified each samples association and the explanatory value of physico-chemical parameters. A non-metric multidimensional scaling (NMDS) (Vegan) plot was used to visualize how environmental variables and grouping factors (Oceanic provinces, and clusters) explained the distribution of viral contigs.

## 3. Results

Bacteriophage taxonomic diversity was inferred from 64 metagenomic and six metaviromic samples collected along nine degree of latitude (37°–28° S), across the EAC and the Tasman Sea during September 2016. In order to better elucidate the bacteriophage diversity, the analysis focussed on a subset of 1770 of the assembled contigs, ranging from 9 kbp to 197 kbp. The water column depth for each sample was categorised as followed: surface (0–30 m), DCM (deep chlorophyll maximum) (60–130 m), deep (500–4000 m). To better elucidate the possible role of AMGs in phage adaptation to the different provinces sampled, we investigated the distribution of the normalized counts of open reading frames for each sample.

### 3.1. Viral Similarity to Previously Published Databases

**Figure 2:**
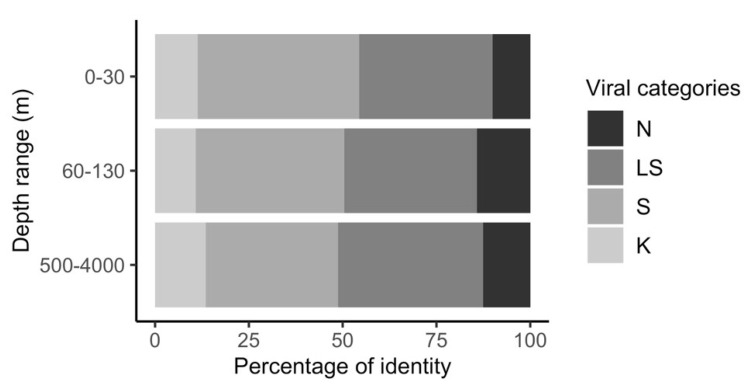
Depth distribution of novel and known bacteriophage contigs. Similarity to previously sequenced viromes and metagenomes was assigned with a BLASTn analysis against the IMG/VR database (2018) using a similarity cut-off of >60% genes with >70% nucleotide identity. Similarity categories: N (novel: shared < 20% genes), LS (low similarity: share > 20% and < 60% genes), S (similar: share >60% and < 80% genes), K (known phage: share > 80% of the genes). Contig abundance was normalized based on read counts.

Genes from the 1770 contigs were screened against the IMG/VR [36,46] databases using BLASTn to assess the degree of similarity of the viral contigs with previously sequenced and assembled viral genomic fragments from other metavirome or metagenomic studies (Figure 2). Approximately half of the contigs shared little similarity (<60% of shared genes) to previously sequenced bacteriophage and could be considered as novel bacteriophage (Figure 2), suggesting that phage that inhabit the aphotic zone are highly underrepresented in current databases. However, a considerable number of the contigs analysed, 202 out of the 1770, were highly similar to previously sequenced bacteriophage (>80% of shared genes) that had been found in different oceanic regions. These widely distributed phages are likely to play an important role in the microbial assemblages and marine ecosystems in general.

### 3.2. Viral Diversity in the Different Oceanic Provinces, Depth Distribution and Comparison between Size Fractions

The sites sampled spanned a wide latitudinal range and presented an extensive environmental variability (Supplementary Table S1). The transect South (in the Tasman Sea) was characterised by lower surface seawater temperature (15–17 °C) and higher nutrient concentrations compared to the EAC drift and the other transects. The EAC sampling sites displayed higher surface seawater temperatures (21–22 °C) and relatively low nutrient concentrations. The mid-latitude transect was sampled longitudinally from the coast, across the EAC, and then into offshore waters. Along this transect, the coastal site displayed high nutrients and surface seawater temperature between 19–20 °C, while samples in the EAC and from farther offshore were characterised by high surface seawater temperatures, but low nutrient concentrations.

**Figure 3:**
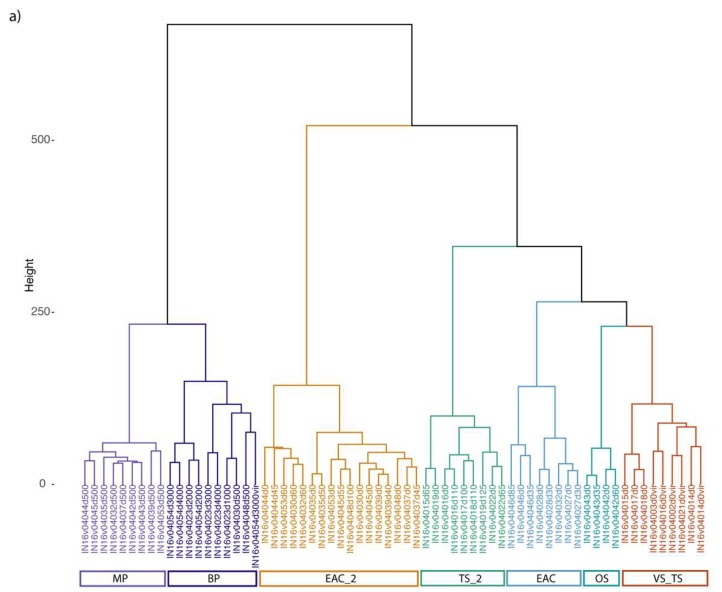

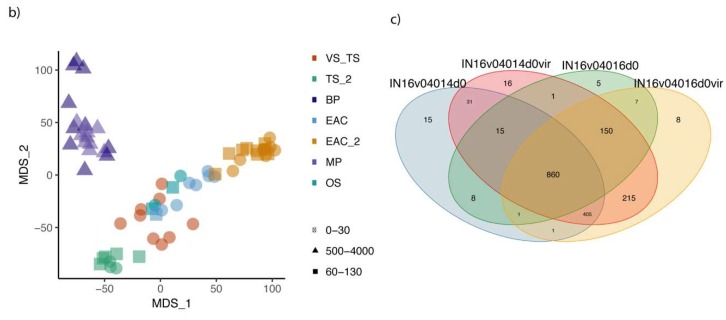
(**a**) Hierarchical clustering of sites based on the normalized abundance of large viral contigs (*n* = 624). A SIMPROF analysis based on Bray–Curtis dissimilarity index was used to identify seven significant clusters. The relative abundance of viral contigs was normalized based on reads count and square root transformed. Sites are color-coded bases on significant clusters. VS_TS = Virome and surface Tasman samples, TS = Tasman Sea surface and DCM, EAC and EAC_2 = EAC, Tr_M and S.N, OS = Offshore samples from Tr_M, MP = Mesopelagic samples (500 m), BP = Bathypelagic samples and virome samples. (**b**) Non-metric multidimensional scaling plot of the bacteriophage diversity observed in the first two axes for all the stations and depths. Samples are color-coded based on hierarchal clusters calculated from the SIMPROF test (Clustsig). Shapes are related to different depth ranges. (**c**) Venn diagram showing the distribution of shared and unique viral contigs in the microbial fraction (>0.2 µm) (INv04014d0, IN16v04016d0) and the viral fraction (<0.2 µm) (INv04014d0vir, IN16v04016d0vir), from the same sites.

A similarity profile test (SIMPROF) identified seven significant clusters of samples based on the relative abundance of sequence reads mapping to distinct bacteriophage contigs (Figure 3a). Cluster analysis showed a clear separation between samples collected in the euphotic zone compared with those from the aphotic zone.

Samples from the euphotic zone grouped in five different clusters. The clusters designated VS_TS includes both surface samples from the Tasman Sea and viriome samples. TS_2 instead grouped just sites from the Tasman Sea. Clusters EAC and EAC_2 and OS grouped site from either the EAC, S.N and Tr_M. The clustering results suggest a distinct partitioning of euphotic zone viral communities between the EAC and Tasman Sea (*p* < 0.001). The deep samples fell into two clusters, corresponding to different depths: mesopelagic MP (depth 500 m) and bathypelagic BP (depth > 500 m). This neat separation of bacteriophage diversity in samples taken from deep vs. photic zone and Tasman Sea vs. EAC was also evident from the non-metric multidimensional scaling (nMDS) analysis (Figure 3b).

In order to understand if the main viral community was comparable in the two fractions (>0.2 and <0.2), the normalized contigs count in each virome sample was compared with the metagenome counterpart. While the difference was significant, for the surface samples the majority of the larger contigs were present in both the fractions (Figure 3c). However, the deep sample (CTD54d3000) reported a higher variability between the metaviriome and metagenome fraction, possibly due to the low number of viral reads recovered from the deep metagenome.

### 3.3. EAC and Tasman Sea Presents Different Viral Communities

**Figure 4:**
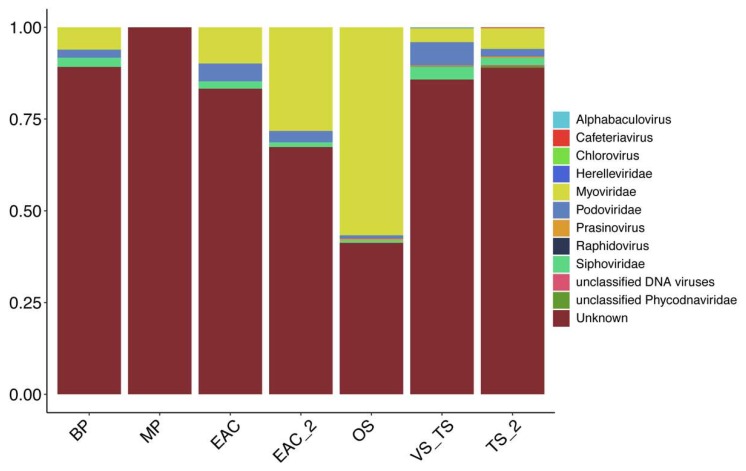
Relative proportion of sequence reads mapped to taxonomically assigned viral contigs at the family level in the different clusters. Taxonomy was assigned with Kaiju against the NCBI virus database (December 2019).

**Figure 5:**
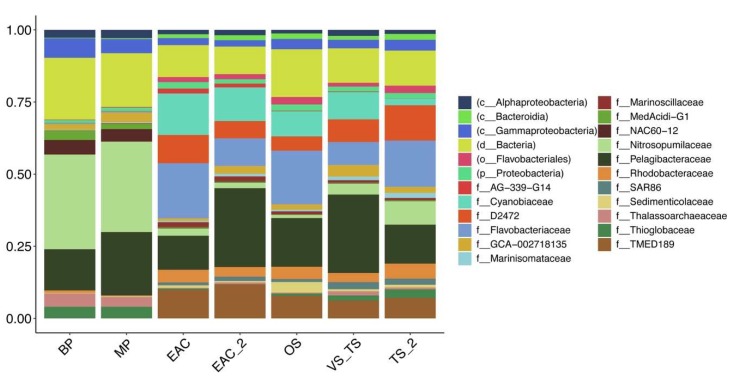
Community composition for the different clusters of the 50 most abundant prokaryotic taxa in the metagenomes represented at the family level. Taxonomy was estimated from SSU rRNA genes (bacteria and archaea), reconstructed from shotgun metagenomes using phyloFlash.

Although more than half of the viral contigs could not be taxonomically assigned, there was a clear difference in the families identified in the different provinces (Figure 4). Virus belonging to the families of Siphoviridae, Podoviridae, and Myoviridae were present in all the clusters from the euphotic zone. However, contigs associated with the Myoviridae, to which a major fraction of bacteriophage species known to infect either *Synechococcus*, or *Prochlorococcus* belongs, were statistically higher in samples from the EAC (Supplementary Figure S1). Consistent with this, the microbial fraction for the EAC clusters showed an increase in the cyanobiaceae family (GTDB database), to which both *Synechococcus* and *Prochlorococcus* belong (Figure 5). Even though Virsorter was optimized especially for the detection of bacteriophage [33], it still recovered the signature of possible abundant eukaryotic viruses. Indeed, the samples from the Tasman Sea clusters showed a higher abundance of Siphoviridae, and also of Prasinovirus, a virus known to infect eukaryotic algae (Supplementary Figure S2). At the species level, the Tasman Sea samples were enriched with *Puneceispirillum* phage, that is known to infect SAR116, Pelagibacter phage, Roseobacter viruses and Cellulophaga phage, while the EAC was dominated by *Synechococcus* and *Prochlorococcus* phage (Supplementary Figure S2).

Samples from the aphotic zone registered the highest percentage on unknown bacteriophage, suggesting once again that both meso- and bathypelagic zone are still highly understudied in term of viral diversity.

The microbial fraction displayed an increase in the relative abundance of the Rhodobacteraceae family, that includes *Roseobacter*, in the Tasman Sea, along with an increase in the SAR86 cluster (d2472 family). Samples collected in the aphotic zone were characterized by a higher relative abundance of archaea, especially the nitrosopumilaceae family.

### 3.4. Functional Profile and Main AMGs in the Different Regions

**Figure 6:**
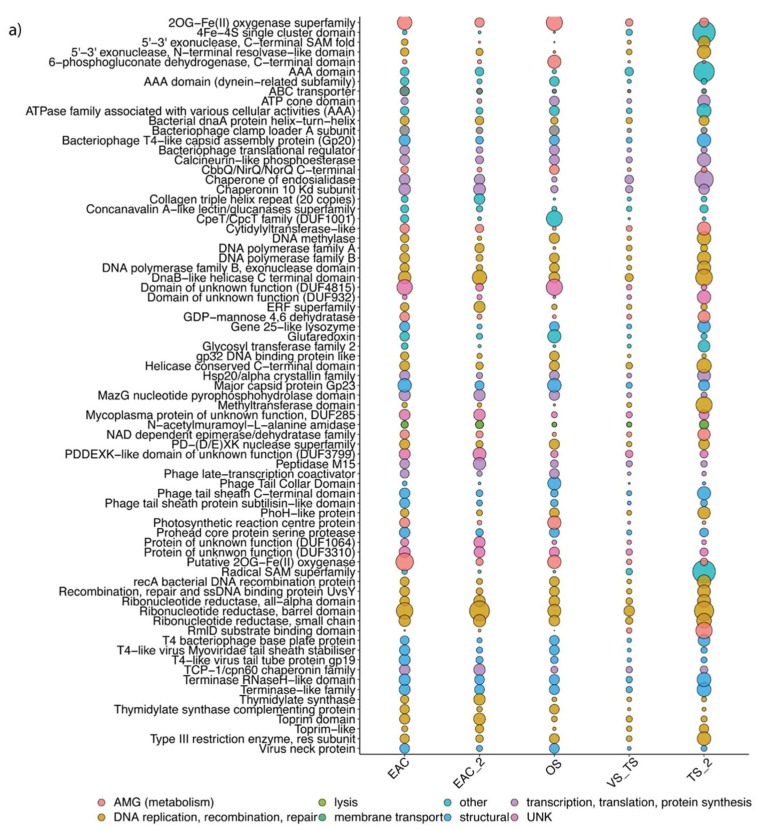

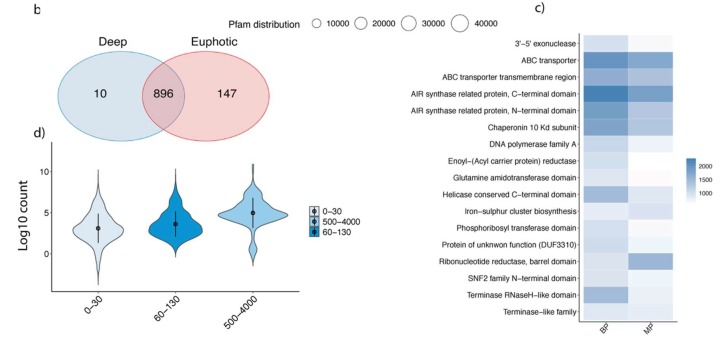
(**a**) Differential distribution of the 80 most abundant Pfam in the different clusters of the euphotic zone. Function was assigned with Interproscan (v.5). Pfam descriptions are colour coded based on corresponding functional categories [38]. Samples are grouped based on hierarchal clusters, calculated using the SIMPROF test (Clustsig). Size of the bubble is relative to Pfam abundance. (**b**) Venn diagram illustrating the differential distribution of shared and unique annotated viral genes between the aphotic and the euphotic zone. (**c**) Differential distribution of the 17 most abundant Pfam in the aphotic zone. (**d**) Log scaled depth distribution of integrase genes from the viral database.

The differences evident in the geographical distribution of bacteriophage sequences were also reflected in the profiles of genes associated with functional roles (assessed by the relative abundance of genes matching Pfams in the different samples). The functional profiles were distinctly different between the surface and deep samples, i.e., photic vs. the aphotic zone, across all sampled sites (Figure 6b).

Structural genes and genes involved in DNA replication and repair were amongst the most abundant in samples collected between 500–4000 m. ABC transporters were the main auxiliary metabolic genes (AMGs) present in deep samples (Figure 6c). Deep samples also displayed a relative increase in genes related to phage lysogenic strategies, especially integrases (Figure 6d), although the observed increased was not statistically significant (ANOVA followed by Tukey’s test in R). However, an analysis of the depth distribution of integrases in the complete gene catalogue from both the microbial and viral fraction did show a significant difference in phage life strategies between the surface and deep samples (*p* < 0.001) (Supplementary Figure S3).

In the photic zone, the most abundant genes encoded deoxyribonucleotide biosynthesis enzymes. EAC samples were characterised by a high prevalence of AMGs usually encoded by phages that infect cyanobacteria. Genes that encode for proteins involved in photosynthetic activity (*psbA*, *psbD*, T-type Phycobiliprotein Lyase) and in nitrogen-related stress (2OG-Fe-oxygenase) were amongst the most common, followed by transaldolase (PPP), MazG which is involved in cell survival under nutritional stress conditions, and multiple peptidase families. The functional profile for the samples collected in the Tasman Sea was characterised by a prevalence of genes that encode for an enzyme related to the radical SAM superfamily (*p* < 0.01), a 4Fe-4S single cluster domain, and also RmID, an enzyme involved in the biosynthesis of the precursor of L-Rhamnose. Furthermore, Tasman Sea samples were enriched with different methyltransferases, glycosyltransferases and pyrophosphohydrolases, some of which were almost absent in the EAC (Figure 6a).

### 3.5. Influence of Environmental Parameters on Viral Communities

**Figure 7:**
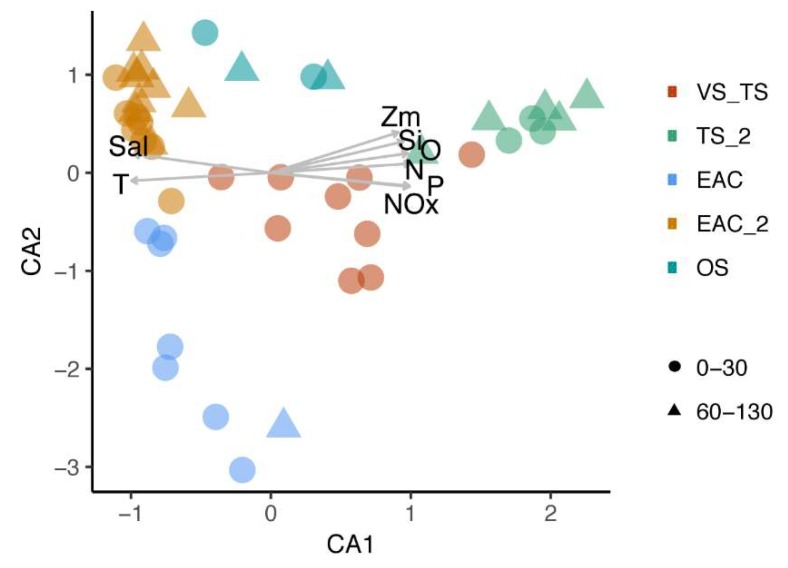
Canonical correspondence analysis (Vegan) plot of the main (1770) virus contigs distribution for the different sites. Samples are colour coded based on cluster assigned with hierarchical cluster analysis (SIMPROF). Salinity—Sal, temperature—T, mix layer depth—Zm, silicate—Si, oxygen—O, nitrite/nitrate—NOx, nitrite—N, phosphate—P.

The variation in viral community composition in the photic zone significantly correlates (*p* < 0.01) with the environmental parameters associated with the different oceanic provinces. Canonical correspondence analysis (CCA) identified temperature (T), salinity (Sal), phosphate (P), nitrite/nitrate (NOx), nitrite (N), silicate (Si) and oxygen (O) as significant parameters that explained 40% of the total variation (Figure 7). EAC and Tasman Sea samples were effectively separated along CCA1, with temperature and salinity being positively correlated with the EAC clusters, and nutrients and O_2_ being positively correlated with the Tasman Sea clusters.

## 4. Discussion

On a daily basis, viruses are thought to be responsible for the infection of up to 20–40% of microbes in the oceans [1], meaning that they can have a substantial impact on the activity of these microbes that play key roles in all the marine biogeochemical cycle.

Here, we carried out the first study of the genetic diversity of the bacteriophage communities for the region encompassed by the EAC and the Tasman Sea. Understanding bacteriophage diversity is an important first step to better understanding their impact on the microbial community dynamics and composition in this region that is undergoing significant climate-related change [18,19]. Although the recent emergence of new generation sequencing technologies has greatly accelerated the discovery of viruses in the marine environment [47,48,49], the majority of the large genomic contigs we obtained (377/624) were still not related to sequences present in existing databases and therefore represent putative novel bacteriophage. This result emphasizes the knowledge gaps that still exist in understanding the diversity of marine bacteriophage, especially for the Southern Hemisphere. This study provides a benchmark dataset to start to understand bacteriophage diversity and their potential role in driving the microbial biogeographic patterns along this region.

Significant differences, both at the taxonomic and functional levels, of the bacteriophage communities along the east coast of Australia were observed. These taxonomic and functional differences could be largely explained by differences in temperature and nutrients characteristics of the different water masses. However, we acknowledge that with the use of Virsorter as a detection tool, and the focus on larger contigs, we possibly overlooked the diversity of the viral community. The specific Virsorter was developed for the identification of either bacterial and archaea infecting viruses and so it would not capture the total diversity of eukaryotic infecting viruses.

### 4.1. Comparisons between Different Size Fractions: How Much Diversity Are We Losing?

Methods to isolate, enrich or separate viruses from marine bacterioplankton assemblages typically involve concentration of biotic material by filtration through different pore size filters to enrich for specific size fractions: e.g., microbial and attached fraction (>0.2 µm) and the viral fraction (<0.2 µm). To assess the recovery of bacteriophage from each method, we compared the diversity of the identified bacteriophage contigs between the two size fractions at four different sites (CTD14, CTD16, CTD21, for surface and CTD54 for deep samples). The viral fraction clustered reasonably well with the microbial fraction collected from the same site (Figure 3a) for the subset of the community analysed (i.e., >9 kbp contigs). However, a pairwise comparison between each virome and the microbial metagenome counterpart showed a difference in the distribution of the bacteriophage contig based on normalized viral reads count (Figure 3c). While the most abundant contigs were present in both the fractions, the metaviromes displayed a higher diversity with the presence of multiple low abundance contigs that were not detectable in the microbial metagenome. This was especially the case for contigs shorter than 15 kpb. For example, at site CTD14, 380 out of 1770 contigs were only detectable in the viral fraction (Figure 3c), of which 288 were smaller than 15 kpb. In contrast, only 25 contigs were statistically more abundant in the microbial fraction. The latter group may represent DNA from phages that were actively infecting bacteria, and thus higher in number in the microbial and attached fraction (>0.2 µm). Despite these differences, here we showed that, at least for surface sites, virus sequences from the microbial fraction could provide a useful starting point to understand the diversity of the main bacteriophage community in seawater.

### 4.2. Ecology of Mesopelagic and Bathypelagic Phages

Bacteriophage from the aphotic zone were almost all classified as unknown, and shared the least similarity with previously known phage (Figure 2), suggesting that the diversity of both mesopelagic and bathypelagic phage is still underrepresented in current databases [50,51,52]. The neat separation of mesopelagic and bathypelagic communities into distinct clusters is in accordance with previous analysis on the global virome, that found similar separation at least in temperate and tropical latitudes [53].

Marine deep ecosystems are characterized by cold temperatures and the limited bioavailability of nutrients to sustain a high microbial metabolism. The genomic content revealed a high number of putative transporters and enzymes related to the de novo synthesis of nucleotides (AIR synthase). The expression of virally-encoded transporters may enhance the uptake of nutrients by the host, to later be used as building blocks for the de novo synthesis of nucleotide and ultimately the assembly of new phage [42]. These results emphasize the potential of viruses to enhance microbial nutrient scavenging, especially in habitats where nutrients are a limiting factor for microbial growth and metabolism.

The relative increase of integrase genes with depth suggests an increase in the potential for a lysogenic lifestyle. This result is strengthened by the comparable trend in the integrase gene depth distribution for both the virome database (Figure 6c) and the total genes from the microbial database (Supplementary Figure S3). A lysogenic lifestyle could be preferable in a situation where the host growth or metabolism is either compromised or slowed down, like deep ecosystems, where there is a possible limitation of resources for virion production. Moreover, host genome size has also been linked with an increase in bacteriophage lysogenic lifestyle [54]. Environments that have less constraints in organism genome size could be more permissive with the accumulation of exogenous DNA, like prophage, and thus favor a bacteriophage lysogenic lifestyle [55]. Deep microbes are indeed usually characterized by a larger genome size [56].

### 4.3. Phage Adapt Their Genomic Content Based on Host and Environmental Conditions

In contrast to deep ecosystems, viral diversity, both at the taxonomic and functional levels in the euphotic zone, presented a marked regional distribution pattern (Figure 6).

Due to the reliance of bacteriophage on their hosts for replication, their abundance is usually tightly coupled to the presence and regional distribution of their specific hosts. Moreover, bacteriophage have been strongly correlated with the succession of their specific host ecotypes [57]. Previous work [25] and concurrent genomic (Figure 5) data showed that cyanobacteria are highly abundant in the EAC; not surprisingly phage thought to infect cyanobacteria predominated in the warm water of the samples collected in the EAC (EAC, EAC_2). Here, the cyanophage possess auxiliary metabolic genes to exploit the cyanobacterial photosynthetic apparatus to boost their production. Putative cyanophage contigs from the EAC were indeed enriched in Pfams involved in photosynthesis, including core photosystem components (psbA, psbD), and phycobiliprotein Lyase (Supplementary Table S2). While *psbA* genes are not essential for phage, they confer a selective advantage in high-light conditions where light damaged cyanobacterial photosystem II machinery can be replaced with functional copies derived from phage genes [13]. T-type phycobiliprotein lyase could provide an advantage for a fast phycobiliprotein assembly during phage infection [58].

Nutrient availability (nitrogen, phosphate, iron) is among the main factors limiting the growth of phytoplankton [59]. Western boundary currents, such as the EAC, are known to be relatively poor in nutrients. Indeed, in our dataset, samples from the EAC were characterised by lower concentrations of nitrogen and phosphorus, compared to those from the Tasman Sea. Genes that encode proteins related to nutrient stress in the cell (2OG-Fe, MazG, PhoH) and nutrient scavenging (phosphate-binding protein, putative ABC transporter) were highly enriched in the EAC viral contigs. MazG, a nucleoside triphosphate pyrophosphohydrolase, has a role in the hydrolysis of ppGpp (Guanosine pentaphosphate). The cellular concentration of ppGpp usually increases when the cell is in a starvation state. By removing ppGpp, the phage encoded MazG could play a role in “reactivating” host metabolism by simulating levels that would normally occur in a nutrient-replete environment, and thus eventually facilitating virus production [60,61]. Finally, *talC*, a gene that encodes an enzyme responsible for the diversion of carbon from the Calvin cycle to the PP pathway [17], was also significantly abundant in the EAC and offshore samples from the north and middle transect. Transaldolases are commonly encoded in cyanophage genomes [17] and the expression of the phage encoded *talC* supposedly leads to an increase in energy available for phage production. Altogether, these results highlight how bacteriophage in the EAC may exploit photosynthetic bacterial communities, by modulating their photosynthetic apparatus to cope with higher light intensity, but also expressing carbon cycle genes that divert energy and carbon towards phage production and nutrient scavenging genes to boost the capture of limiting nutrients [17,42].

While the EAC is usually dominated by prokaryotic primary producers, and hence the phage that infect them, the colder and more mesotrophic Tasman Sea is enriched with eukaryotic primary producers instead of cyanobacteria [24,25,62]. Taxonomic classification of the microbial metagenome showed an increase in the Rhodobacteraceae family along the Tasman Sea; *Roseobacter* belongs to this family, and this is consistent with our observed decrease in cyanophage and relative increase in Cellulophaga and Roseobacter phages, but also Puneceispirillum phages.

Both Cellulophaga and Roseobacter species are usually found in association with marine algae; the latter comprise a group of bacteria that have an important role in the oceanic sulphur cycle through the degradation of DMSP produced by marine algae [63]. In contrast to EAC bacteriophage, Tasman Sea bacteriophage displayed a different pool of AMGs that could support phage production (Figure 6). To modulate their metabolisms, the main AMGs encoded for Fe-S cluster protein; Radical SAM superfamily protein, and glycosyltransferases. Iron-sulphur cluster proteins are widespread in viral contigs in the photic zone of the ocean [52] and are involved in a variety of processes that include electron transfer, catalysis and regulatory processes and oxygen-iron sensing. They are also the main prosthetic group of the radical SAM, a superfamily of enzymes that are involved in the biosynthesis of vitamins, coenzymes, antibiotics and also serve as an oxidizing agent in DNA repair [64]. Glycosyltransferase like family genes are usually encoded by lytic phage to protect their DNA during infection from host enzymes, but in lysogenic phage could be involved in modulating the bacterial cell surface [65], possibly affecting susceptibility to infection by other bacteriophages. These data could be further used to look into the distribution of other specific viral metabolic genes (e.g. virulence factors; toxins, antibiotic-resistant genes, etc.) known to be implicated in host fitness [66].

## 5. Conclusions

In conclusion, using both a genome and gene-centric approach, we provide the first insights into the distribution and functional diversity of the main bacteriophage inhabiting the EAC and the Tasman Sea. Meso- and bathypelagic bacteriophages are still mostly unknown, but they possibly represent one of the major players in deep nutrient recycling. Moreover, phage communities from the aphotic zone were similar across a wide latitudinal gradient, but different from the phage found in the euphotic zone. The euphotic zone instead presented different bacteriophage communities associated with either the EAC or Tasman Sea. The difference in bacteriophage communities could possibly be explained by the regional distribution of their specific host, such as cyanophage in the EAC. The genomic content associated with the different environments provide an indication as to how phage adapt their specific repertoire of auxiliary metabolic genes (AMGs) to better exploit their host, based on a change in nutrient bioavailability and temperature.

## Supplementary Materials

The following are available online at https://www.mdpi.com/1999-4915/12/3/317/s1, Supplementary Figure S1: Distribution of the main bacteriophage family. Supplementary Figure S2: Distribution of the main bacteriophage species. Supplementary Figure S3: Distribution of integrase genes. Supplementary Table S1: Environmental parameters of the location where the samples were collected. Supplementary Table S2: Reads mapping statistics. Supplementary Table S3: Functional categories in the virome selected contigs and in the total virome database.
