# Supplementary material for: Investigating the Diversity of Marine Bacteriophage in Contrasting Water Masses Associated with the East Australian Current (EAC) System
Source: Viruses. 2020 Mar 16;12(3):317. doi: 10.3390/v12030317 (PMC7150976; doi:10.3390/v12030317)
Supplement: Supplementary file 1 [file viruses-12-00317-s001.zip › supplementary/Supplementary_Virus.docx]

**Investigating the diversity of marine bacteriophage in contrasting water masses associated with the East Australian Current (EAC) system.**

A.Focardi, M.Ostrowski, K.Gooseen, M.Brown, I.Paulsen

Supplementary figures


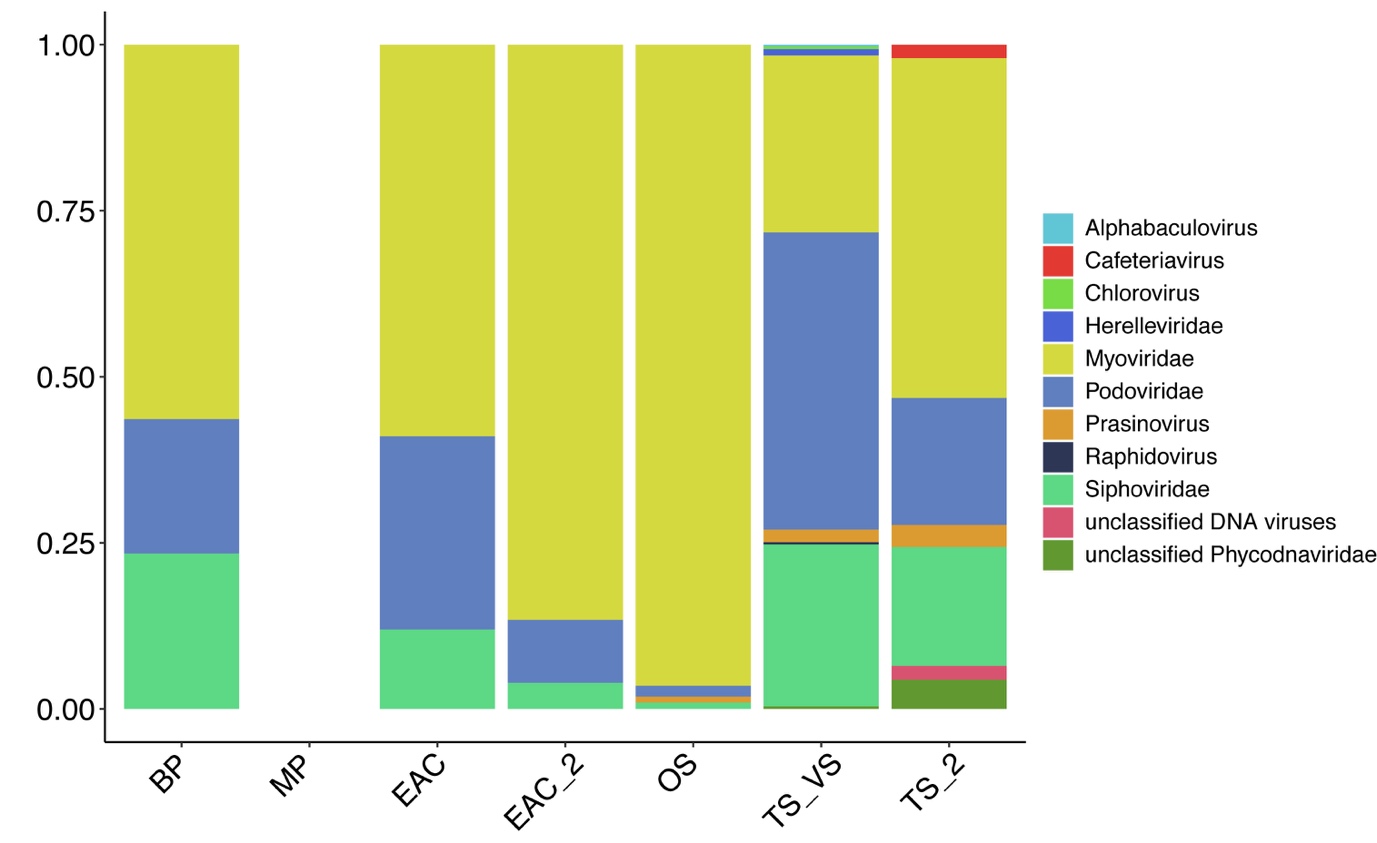


**Figure 1.** Relative distribution of the known main bacteriophage family in the different clusters identified with cluster analysis in R.


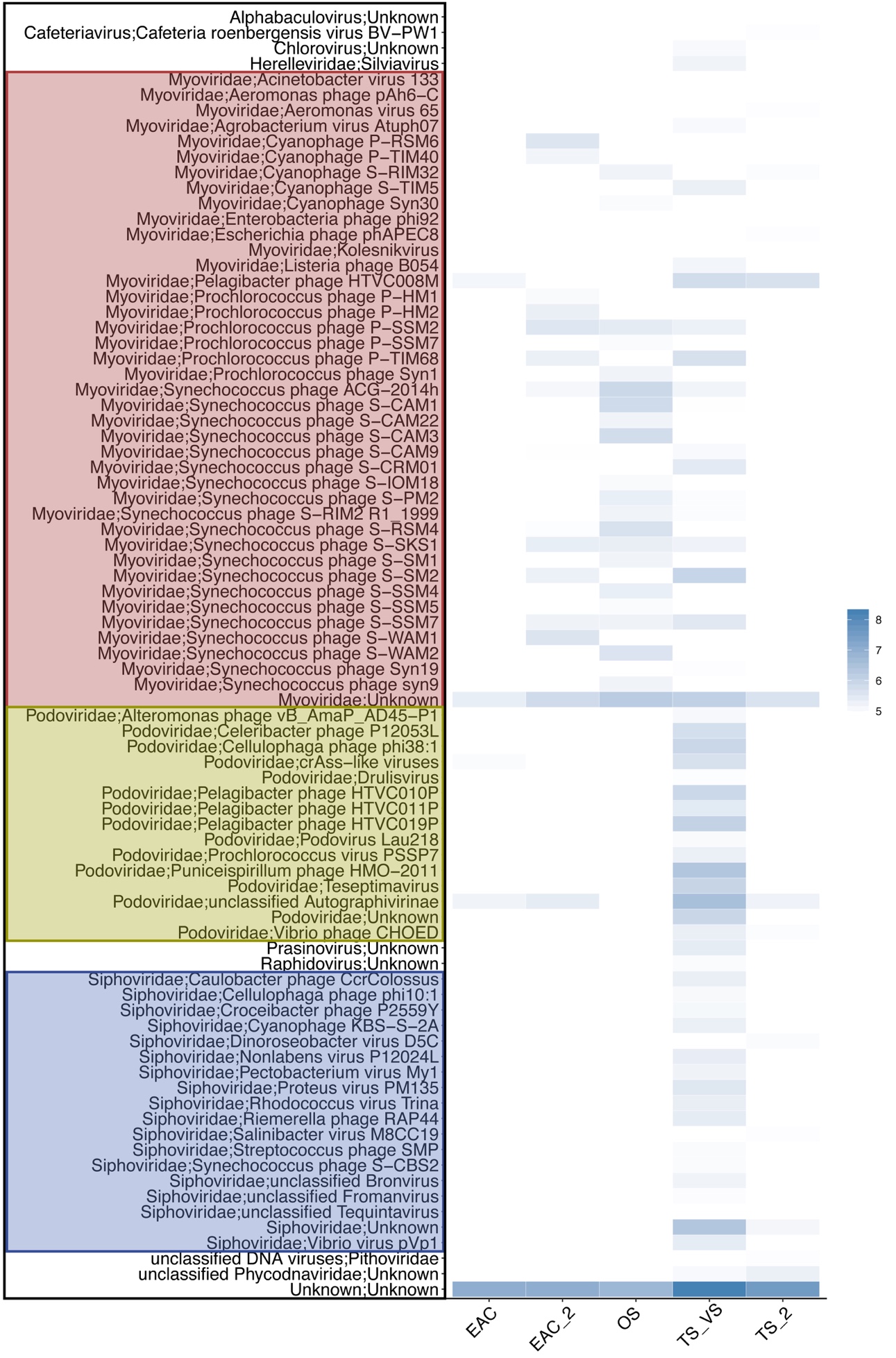


**Figure 2.** Distribution of the main bacteriophage species in the different clusters identified with cluster analysis in R. Species are grouped and colour coded based on family affiliation ( Myoviridae : red, Podoviridae : green, Siphoviridae: blue. Species count are log10 transformed.

**Figure 3.** Depth distribution of the integrase genes in the microbial gene database. P-value was calculated by a pairwise analysis of variance (ANOVA) followed by a Tukey test.
